# Traumatic Brain Injury Dysregulates MicroRNAs to Modulate Cell Signaling in Rat Hippocampus

**DOI:** 10.1371/journal.pone.0103948

**Published:** 2014-08-04

**Authors:** Liang Liu, Tingyi Sun, Zilong Liu, Xiaorui Chen, Lili Zhao, Guoqiang Qu, Qingjie Li

**Affiliations:** 1 Department of Forensic Medicine, Tongji Medical College of Huazhong University of Science and Technology, Wuhan, China; 2 Key Laboratory of Evidence Science, China University of Political Science and Law, Ministry of Education, Beijing, China; 3 Department of Internal Medicine, The University of Texas Medical Branch at Galveston, Galveston, Texas, United States of America; Universidade de São Paulo, Brazil

## Abstract

Traumatic brain injury (TBI) is a common cause for cognitive and communication problems, but the molecular and cellular mechanisms are not well understood. Epigenetic modifications, such as microRNA (miRNA) dysregulation, may underlie altered gene expression in the brain, especially hippocampus that plays a major role in spatial learning and memory and is vulnerable to TBI. To advance our understanding of miRNA in pathophysiological processes of TBI, we carried out a time-course microarray analysis of microRNA expression profile in rat ipsilateral hippocampus and examined histological changes, apoptosis and synapse ultrastructure of hippocampus post moderate TBI. We found that 10 out of 156 reliably detected miRNAs were significantly and consistently altered from one hour to seven days after injury. Bioinformatic and gene ontology analyses revealed 107 putative target genes, as well as several biological processes that might be initiated by those dysregulated miRNAs. Among those differentially expressed microRNAs, miR-144, miR-153 and miR-340**-**5p were confirmed to be elevated at all five time points after TBI by quantitative RT-PCR. Western blots showed three of the predicated target proteins, calcium/calmodulin-dependent serine protein kinase (CASK), nuclear factor erythroid 2-related factor 2 (NRF2) and alpha-synuclein (SNCA), were concurrently down- regulated, suggesting that miR-144, miR-153 and miR-340**-**5p may play important roles collaboratively in the pathogenesis of TBI-induced cognitive and memory impairments. These microRNAs might serve as potential targets for progress assessment and intervention against TBI to mitigate secondary damage to the brain.

## Introduction

Traumatic brain injury (TBI) is a common trauma resulting from industrial accidents, traffic accidents, falls, violence and battlefield. An estimated 10 million people worldwide suffer from TBI every year [1]–[3]. It contributes to a substantial number of deaths and cases of permanent disability, and many victims have functional impairments such as motor and sensory dysfunction, and cognitive deficits including impaired learning and memory [4]. The hippocampus, a key brain structure for cognition, is particularly vulnerable to TBI. The earliest and most severe neuropathological changes occur in the hippocampus after TBI [5], [6]. However, molecular mechanisms underlying hippocampal alterations and cognitive impairments following TBI remain elusive. Identification of specific genes and signal transduction pathways directly involved in TBI is essential for development of novel therapeutic strategies.

Small non-coding single-stranded RNA molecules composed of 20–25 nucleotides, known as microRNAs (miRNAs), post-transcriptionally regulate target mRNAs through the 3′-UTR [7]–[9]. In animal cells, miRNAs are more commonly base paired with the target mRNA and inhibit protein translation. The binding of miRNAs to complementary mRNA can degrade the mRNA and therefore terminate protein translation. Or miRNA can inhibit the reading of the 5′-cap and prevent translation. Recently, it has been shown that miRNAs can also activate gene transcription [10] and enhance mRNA translation [11]. It is estimated that 20–30% of human protein-coding genes are directly regulated by miRNAs [12]. Not surprisingly, miRNAs have recently been linked to various diseases. Numerous miRNAs have been demonstrated to highly express in the mammalian central nervous system and considered as the key modulators of cell differentiation, proliferation, apoptosis, neuronal development, neuroprotection, synaptic plasticity, etc. [13], [14]. Differentially expressed miRNAs were observed in hippocampus of rodents after TBI [15], [16]. However, the involvement of miRNAs in TBI-induced pathophysiological alterations in hippocampus and the contribution of miRNAs to the TBI-induced cognitive impairments remain largely unknown.

In the present study, we assessed dynamic miRNA expression profiles in rat ipsilateral hippocampus after experimental TBI, and identified 156 miRNAs, among which 10 were significantly altered at all five time points. After predication of 107 putative target genes by using three online programs, we carried out bioinformatic and gene ontology (GO) analyses to identify the related biological processes/terms. TBI-induced up-regulation of miR-144, miR-153 and miR-340-5p were further confirmed by quantitative reverse transcriptase-polymerase chain reaction (qRT-PCR), and three of their target proteins, calcium/calmodulin-dependent serine protein kinase (CASK), nuclear factor erythroid 2-related factor 2 (NRF2) and alpha-synuclein (SNCA) were found to be concurrently suppressed. Our findings suggest that miRNAs, especially miR-144, miR-153 and miR-340-5p, are important mediators in pathophysiological processes after TBI and might serve as potential targets for intervention against brain damage after TBI.

## Materials and Methods

### Animals and Surgical Procedures

Adult Sprague-Dawley (SD) rats of either gender weighing 200–250 g were purchased from the Laboratory Animal Center of Tongji Medical College, Huazhong University of Science and Technology (HUST), Wuhan, China. Animals were housed in a controlled temperature environment under a 12 h light/dark cycle, with access to food and water ad libitum. Animal use and all experimental procedures were on conformity with the regulation of “Control Ordinance of Laboratory Animals of Hubei Province, China”, and approved by Animal Care and Use Committee of Huazhong University of Science and Technology. Precautions were taken to minimize suffering (see anesthesia procedures below) and the number of animals used in each experiment.

Ninety SD rats were randomly divided into six groups (n = 15 each): one sham-operated group and five TBI groups according to the time points of experiments: 1 hour, 1 day, 3 days, 5 days and 7 days post injury. A unilateral controlled cortical impact (CCI) brain injury was performed under aseptic conditions as described in previous studies [15], [17], [18]. Injury severity can be precisely controlled by adjusting the parameters of CCI, such as impact velocity, depth and duration. The parameters in the present study were set to induce moderate TBI (a Glasgow Coma Scale of 9–12). In brief, under anesthesia by intraperitoneal injection of 10% chloral hydrate (3 ml/kg) [19], [20], rats were mounted in a stereotaxic device with heads held in a horizontal plane. Body temperature was monitored and maintained at 37°C using a rectal probe and a heating pad. The surgical area of the head was shaved and disinfected with iodine and 75% alcohol before the incision along the midline. Following exposure of the parietal, a 4 mm×4 mm unilateral craniectomy was performed with the frontal edge 1 mm posterior to bregma and the medial edge 2 mm lateral to the midline, using a high-speed dental drill. Precautions were taken to keep the integrity of the dura mater. Each rat received a single contusion on the left temporoparietal cortex using an electromagnetic piston at a velocity of 3.5 m/sec, duration of 200 ms and depth of 2 mm. Then the incision was sutured and antibiotic ointment was applied, the animals were returned to their home cages after recovery from anesthesia. Sham rats received the same procedures but no contusion.

TBI rats of different groups were euthanized by decapitation 1 hour, 1 day, 3 days, 5 days or 7 days post injury, whereas sham rats were euthanized 1 hour after surgery. Two rats from each group were selected randomly for hematoxylin and eosin (H&E) staining, three rats were used to examine the ultrastructure of the injured hippocampal synaptic organization (CA1 region) by transmission electron microscopy (TEM, Tecnai-G212, FEI Company, Netherlands), and five rats were used for terminal deoxynucleotidyl transferase-mediated dUTP nick end labeling (TUNEL) assay. The remaining five rats of each group were euthanized by decapitation, and ipsilateral hippocampus was immediately dissected under ice-cold artificial cerebrospinal fluid, placed into cryopreservation tubes, flash frozen and stored in liquid nitrogen for molecular studies.

### Total RNA Extraction

Frozen hippocampus was pulverized in liquid nitrogen and total RNA was isolated by using miRNeasy Mini Kit (Qiagen, Valencia, CA) according to the manufacturer’s instructions. RNA quality and quantity were measured using NanoDrop ND-1000 spectrophotometer (Nanodrop Technologies, USA) ensuring ratios of A_260 nm_/A_280 nm_ ∼2 and A_260 nm_/A_230 nm_ ≥1.8. RNA Integrity was determined by agarose gel electrophoresis.

### MicroRNA Microarray Assay

MicroRNA samples were labeled using the miRCURY Hy3/Hy5 Power labeling kit (Exiqon, Vedbaek, Denmark). The Hy3-labeled samples were hybridized on the miRCURY LNA Array (v.18.0) (Exiqon) according to the user’s manual. Following hybridization, the slides were washed several times using wash buffer kit (Exiqon), and finally dried by centrifugation at 400 rpm for 5 min. Next the slides were scanned using the Axon GenePix 4000B microarray scanner (Axon Instruments, Foster City, CA) and imported into GenePix Pro 6.0 software (Axon Instruments) for grid alignment and data extraction.

The data were analyzed by subtracting the background and then normalizing the signals using a LOWESS (Locally-Weighted Regression) filter. The miRNA transcript was considered as reliably detectable only if the signal intensity was greater than 3 times the background standard deviation, the spot coefficient of variation was<0.5, and at least 50% of the repeated probe signals were above the detection level.

### MicroRNA Targets Prediction and GO Analysis

A subset of differentially expressed miRNAs was selected based on data obtained from microarray analysis and statistical analysis of repeated experiments. Three online prediction programs, Miranda (http://www.microrna.org/microrna/home.do), MicroCosm Targets Version 5 (http://www.ebi.ac.uk/enright-srv/microcosm/htdocs/targets/v5/) and miRDB (http://mirdb.org/miRDB/) were used to identify target genes potentially regulated by those miRNAs. Target genes predicted by all three programs were retained for further analysis. Functional annotations of target genes were analyzed by using Gene Ontology (GO) method.

To assess the statistical significance of GO slim term representations in the predicted miRNA target gene data set compared with what would be expected by chance from the population of all genes in the rat genome, we calculated *P* values from the hypergeometric distribution. A GO slim term was considered over represented within the predicted target genes if the corrected *P* value was<0.01.

### Quantitative Reverse Transcriptase-Polymerase Chain Reaction

qRT-PCR was used to confirm the microarray results for selected miRNAs. Equal amount of total RNAs were transcribed into cDNAs using miRNA-specific RT primers. Real-time PCR was performed with the ABI PRISM 7900 system (Applied Biosystems, Foster City, CA) using miRNA-specific primer and fluorescently labeled probe. U6 was used as an endogenous control and a no-template reaction as a negative control. The relative quantification in miRNA expression was determined using the 2^−ΔΔCT^ method with U6 as a normalizer.

### Western Blotting

Hippocampal tissues were homogenized on ice in ice-cold lysis buffer containing 137 mM NaCl, 20 mM Tris-HCl (pH 8.0), 10% glycerol, 1% NP-40, 10 mg/mL aprotinin, 1 mM PMSF, 1 mg/mL leupeptin, and 0.5 mM sodium vanadate. Equal amounts of total proteins (30 µg) were loaded into each well of the SDS-PAGE (8–12%) gels, separated by electrophoresis and then transferred to PVDF membranes. After blocking with 5% skimmed milk for 1 hour at room temperature, blots were probed with primary antibody at 4°C overnight. Membranes were thoroughly washed and incubated with horseradish peroxidase (HRP)-conjugated secondary antibody for 1 hour at room temperature. ECL Western blotting detection reagents (Pierce Biotechnology) were used to visualize blots. Primary antibodies are as follows: anti-CASK antibody (1∶1000, Sigma Aldrich, St Louis, MO), anti-SNCA antibody (1∶5000, Abcam, Cambridge, MA), anti-NRF2 antibody (1∶1000, Abcam), anti-procaspase 3 antibody (1∶500, Proteintech, Chicago, IL), anti-cleaved caspase 3 antibody (1∶500, Bioword Technology, Minneapolis, MN) and anti-β-Actin antibody (1∶5000, Abcam).

### H&E Staining

All histological procedures were uniform for sham and experimental group rats. After decapitation, the whole rat brain was carefully exposed, dissected out and fixed in 4% paraformaldehyde for 24 h. It was then dehydrated in ethanol, defatted in xylene, and embedded in paraffin. Sections were cut on a rotary microtome (Leica RM2235, Germany) at 5-micron thickness and stained with H&E according to standard procedure, and mounted onto slides. The hippocampal CA1 region was studied under a light microscope.

### Tunel Assay

Under deep anesthesia, the rats were transcardially perfused with 100 mL of 0.9% saline and followed by 200 mL of freshly prepared 4% paraformaldehyde in 0.1 M phosphate buffer (pH 7.4). The left hemi-brains were carefully removed and post-fixed in 4% paraformaldehyde at 4°C for 24 h. The tissues were subsequently embedded in paraffin and sections were serially cut at 5 µm.

In situ TUNEL method was used to recognize apoptotic cells by identification of DNA fragmentation according to the manufacturer's instruction (Roche Applied Science, Mannheim, Germany). Briefly, sections were deparaffinized, dehydrated using xylene and ethanol, rehydrated in phosphate-buffered saline, and then incubated with freshly diluted proteinase K at room temperature for 15–30 min. After washing with 1% Triton X-100 for 8 min and rinsing with PBS, slices were subsequently incubated with 50 µl of TUNEL mixture for 60 min at 37°C, and then incubated with 50µl of converter–POD solution for 30 min at 37°C. After rinsing again with PBS, 50 µl diaminobenzidine (DAB) was added in the presence of H_2_O_2_ for 10 min at 15°C–25°C to visualize TUNEL-positive nuclei.

The number of TUNEL-positive neurons was counted in 4 microscopic fields of each slide at a 400X magnification. The mean number of apoptotic cells in the CA1 area of hippocampus was used for each animal.

### Transmission Electron Microscopy

Hippocampal synaptic organization (CA1 region) was cut into 1 mm^3^ blocks, fixed in 4% glutaraldehyde for 48 h, and post-fixed in 1% osmium tetroxide for 1 h. The blocks were then dehydrated in an ethanol-acetone series (50%, 70%, 90% ethanol, 90% ethanol and acetone, 90%, 100% acetone), infiltrated with resin (50% resin in acetone and then 100% resin), and then incubated at 80°C for 10 h. Cross sections were cut with an Leica ULTRACUT UCT ultramicrotome (Leica Microsystems GmbH, Wetzlar, Germany). Ultrathin sections were examined with a transmission electron microscope (TEM, Tecnai-G212, FEI Company, Netherlands).

### Statistical Analysis

Obtained data were compared using ANOVA analysis followed by Post hoc analysis for multiple groups and Student’s *t*-test for two groups. Pearson’s analysis was used to measure the correlation between microRNAs. A *P* value <0.05 was considered to be statistically significant.

## Results

### miRNA Expression Profile in Ipsilateral Hippocampus after TBI

**Figure 1:**
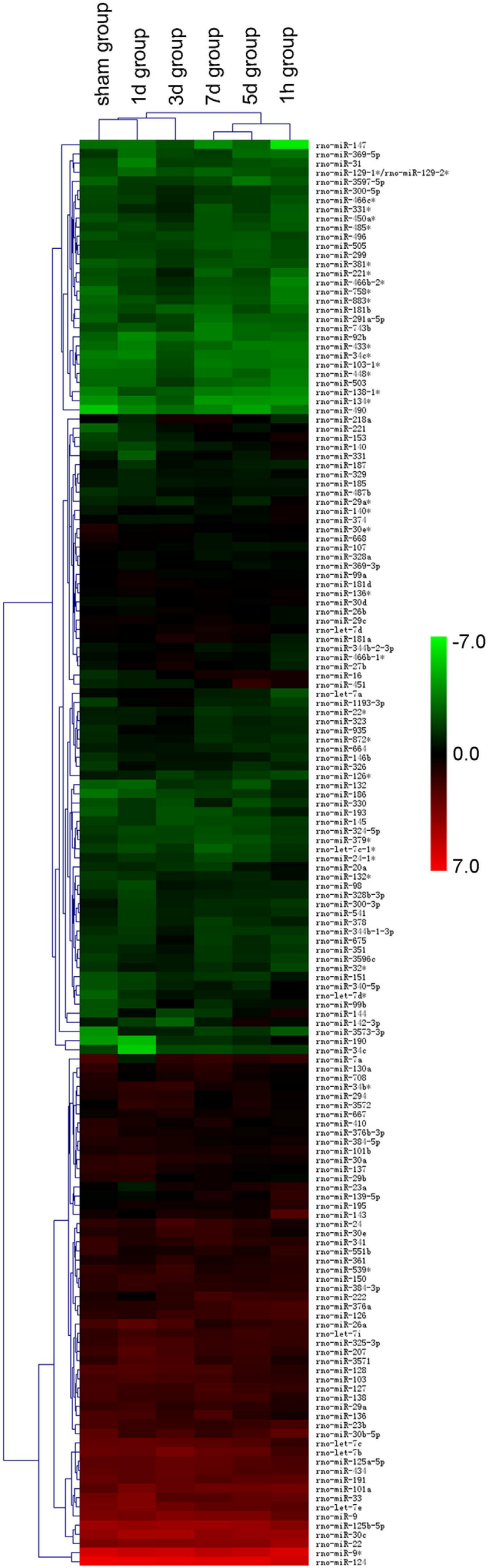
Hierarchical cluster analysis of miRNA array data. Each column represents a sample, and each row represents a single microRNA. The miRNA clustering tree is shown on the left, and the sample clustering tree appears at the top. The color scale shown on the right illustrates the relative expression level of a miRNA in certain slide: green indicates negative values (down-regulation), representing low relative expression levels; red indicates positive values (up-regulation), representing high relative expression levels; black indicates zero (no change). The sham control group and five TBI groups were clustered together in pairs.

Following CCI with fine-tuned parameters according to previous studies, a moderate TBI model was successfully established. Only 2 out of 75 TBI rats died of injury. SD rats received sham surgery were euthanized for brain tissue collection one hour later and animals subjected to TBI were euthanized one hour, one day, three days, five days or seven days after injury. Total RNA was extracted from ipsilateral hippocampus and RNA quality was assessed. To identify dysregulated miRNAs by TBI, miRNA expression profiling was carried out using miRCURYTM LNA Arrays. A total of 30 sets of data from 6 groups (five rats each) were generated. A dendrogram of a Hierarchical Clustering analysis of the results from sham and five TBI groups is shown in Figure 1. Five sets of data from each group were combined for significance analysis of microarrays. Since we used a single channel to scan the array, the data obtained from each group should be compared with the data of sham group to identify differentially expressed miRNAs.

After filtering out miRNAs that were undetectable, 156 miRNAs were reliably detected in sham and five TBI groups. Out of the 156 miRNAs identified, 60, 41, 81, 52 and 60 miRNAs were up-regulated more than 1.5-fold, while 45, 31, 11, 41 and 35 miRNAs were down-regulated more than 1.5-fold at 1-hour, 1-day, 3-day, 5-day and 7-day post-injury, respectively, compared with sham group. There were 10 miRNAs that were consistently up−/down-regulated at least 1.5-fold at all five time points after TBI (Table 1), among which miR-142-3p, -144, -340-5p, -674-5p, -153, -186, -190, -132* and -138-1* were up-regulated, whereas let-7b was down-regulated.

**Table 1:** miRNAs altered by more than 1.5-fold at all five time points post-injury, compared with sham group.

	Time points after TBI				
miRNA	1 hour	1 day	3 days	5 days	7 days
rno-miR-142-3p	1.5173	0.4983	0.2421	2.4795	1.5004
rno-miR-144	8.5134	3.8484	1.5218	4.0641	1.6774
rno-miR-340-5p	4.5029	1.5052	2.4332	2.0825	3.0379
rno-miR-674-5p	18.0381	1.5002	1.5113	6.8001	7.7527
rno-miR-153	5.9797	1.8277	2.1506	3.5749	3.6636
rno-miR-186	4.4307	1.7390	2.3720	3.0968	2.9151
rno-miR-190	17.5808	0.5279	7.7443	9.6853	7.5148
rno-miR-132*	2.2799	1.5079	1.7190	1.5172	1.5322
rno-let-7b	0.4662	0.5721	0.3427	0.4089	0.4931
rno-miR-138-1*	1.5031	2.6835	2.0158	1.5404	1.5019

### Prediction of miRNA Targets and GO Analysis

**Figure 2:**
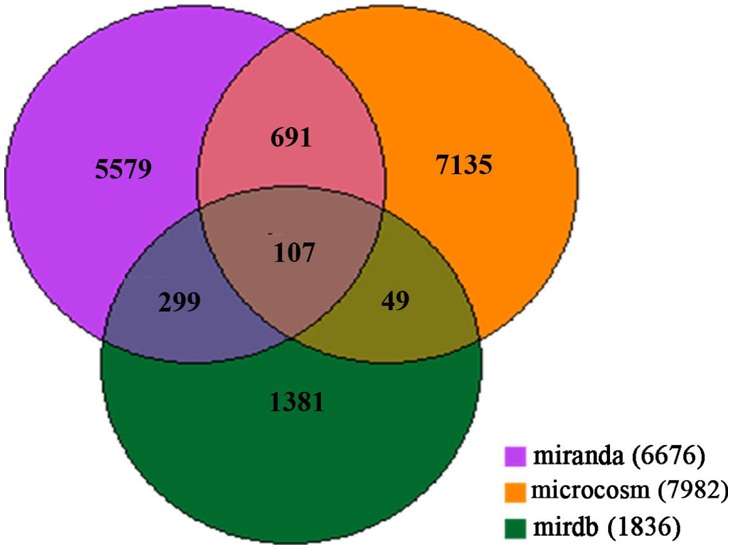
Venn diagram demonstrating 107 putative target genes appeared in all three prediction data set.

**Figure 3:**
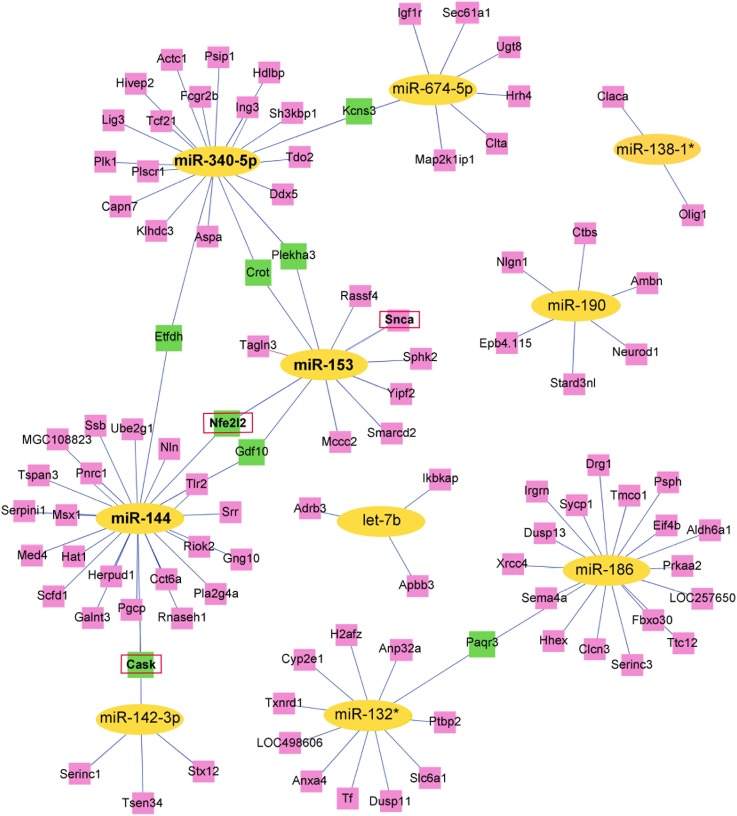
Target genes of 10 miRNAs that were consistently up-regulated or down-regulated at all five time points post TBI. Genes marked in PINK are targeted by a single miRNA, and the GREEN annotations are regulated by two miRNAs.

miRNA target prediction was performed for all ten miRNAs that were altered at all five time points post TBI (Table 1). Three online programs, Miranda (http://www.microrna.org/microrna/home.do), MicroCosm Targets Version 5 (http://www.ebi.ac.uk/enright-srv/microcosm/htdocs/targets/v5/) and miRDB (http://mirdb.org/miRDB/), predicted 6676, 7982 and 1836 target genes, respectively. A Venn diagram (Figure 2) illustrated the intersection among the three prediction data sets, finding a total of 107 target genes that are potentially regulated by those 10 miRNAs. Based on this data set, we constructed a gene interaction network for those 107 genes (Figure 3).

**Figure 4:**
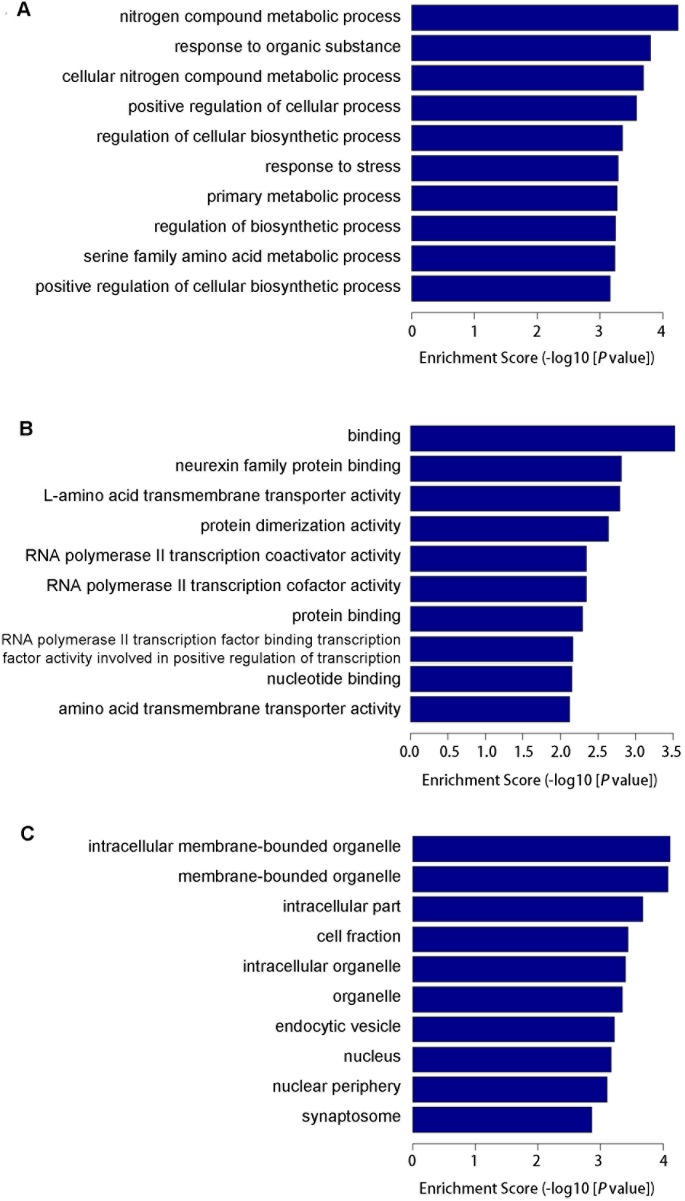
Classifications of predicted target genes according to their gene ontology (GO) annotation terms within biological process (A), molecular function (B) and cellular component (C) ontologies, respectively. These GO terms showed a statistically significantly enriched representation. Enrichment Score of the GO equals (-log10 [P-value]).

For further analyses of target genes, we downloaded functional annotations for the 107 target genes using GO enrichment analysis. We obtained the GO terms annotating the target genes from the GO database, and then performed the enrichment analysis for each GO term by conditional hypergeometric test. Gene function annotations were classified into three categories: biological process (BP), molecular function (MF) and cellular component (CC), and 273, 52 and 52 GO terms were identified for these three categories, respectively. Top ten enriched GO terms of each category according to the enrichment score of the GO terms are shown in Figure 4. Target genes involved in the top ten enriched GO terms of BP, MF and CC ontology are listed in tables 2, 3 and 4, respectively. The number of target genes that were regulated by each miRNA is shown in tables 5, 6 and 7 according to GO terms of BP, MF and CC ontology. A large number of target genes involved in enriched GO terms were targeted by miR-144 and miR-340-5p. In addition, miR-153 is known for its involvement in Parkinson disease (PD) and Alzheimer's disease (AD), so further experiments were focused on these three miRNAs.

**Table 2:** Target genes and enriched GO terms in the biological process ontology.

GO.ID	Term	Genes
GO:0006807	Nitrogen CompoundMetabolic Process	LIG3,PLK1,HHEX,MSX1,TCF21,DDX5,PSIP1,MED4,H2AFZ,CTBS,TXNRD1,ACTC1,ALDH6A1,RNASEH1,XRCC4,HAT1,ANP32A,HIVEP2,OLIG1,PRKAA2,NFE2L2,SMARCD2,PNRC1,ING3,IKBKAP,PTBP2,SSB,SRR,PSPH,TDO2,SPHK2,ADRB3,MCCC2,TF,NEUROD1,SNCA,TLR2,IGF1R,CASK,PLSCR1
GO:0010033	Response to OrganicSubstance	TLR2,HERPUD1,SNCA,PLA2G4A,SLC6A1,NEUROD1,CYP2E1,CROT,TF,SRR,MAP2K1IP1,HHEX,NFE2L2,PRKAA2,PSPH,SEC61A1,PLSCR1,ACTC1,FCGR2B,MSX1,GDF10,CASK
GO:0034641	Cellular NitrogenCompound MetabolicProcess	LIG3,PLK1,HHEX,MSX1,TCF21,DDX5,PSIP1,MED4,H2AFZ,TXNRD1,ACTC1,ALDH6A1,RNASEH1,XRCC4,HAT1,ANP32A,HIVEP2,OLIG1,PRKAA2,NFE2L2,SMARCD2,PNRC1,ING3,IKBKAP,PTBP2,SSB,SRR,PSPH,TDO2,ADRB3,MCCC2,TF,NEUROD1,SNCA,TLR2,IGF1R,CASK,PLSCR1
GO:0048522	Positive Regulation ofCellular Process	SNCA,TLR2,TF,SERINC3,PLK1,ADRB3,PLA2G4A,SPHK2,PRKAA2,NFE2L2,NLGN1,TXNRD1,SLC6A1,NEUROD1,DDX5,MED4,HHEX,IGF1R,MSX1,MAP2K1IP1,ING3,GDF10,CASK,PLSCR1,TCF21,PSIP1,XRCC4,ASPA,FCGR2B
GO:0031326	Regulation of CellularBiosynthetic Process	PLK1,HHEX,MSX1,TCF21,HAT1,ANP32A,HIVEP2,OLIG1,PRKAA2,NFE2L2,SMARCD2,PNRC1,ING3,PSIP1,IKBKAP,MED4,ADRB3,SNCA,TF,NEUROD1,DDX5,PLA2G4A,TLR2,IGF1R,CASK,PLSCR1
GO:0006950	Response to Stress	TF,SCFD1,TLR2,FCGR2B,HERPUD1,PLSCR1,XRCC4,HRH4,TXNRD1,ETFDH,PSIP1,PLA2G4A,ADRB3,HHEX,NFE2L2,PLK1,SNCA,SEC61A1,PRKAA2,PGCP,IRGM,IGF1R,LIG3,CASK
GO:0044238	Primary MetabolicProcess	LIG3,PLK1,HHEX,MSX1,TCF21,DDX5,PSIP1,SNCA,MED4,H2AFZ,UBE2G1,CTBS,TXNRD1,ACTC1,ALDH6A1,RNASEH1,XRCC4,HAT1,ANP32A,HIVEP2,OLIG1,PRKAA2,NFE2L2,SMARCD2,PNRC1,ING3,IKBKAP,PTBP2,SSB,IGF1R,RIOK2,DUSP13,PGCP,NLN,SRR,PSPH,TDO2,CROT,SERINC1,CYP2E1,SERINC3,PLSCR1,SPHK2,UGT8,ADRB3,TLR2,TF,HDLBP,PLA2G4A,MCCC2,NEUROD1,GALNT3,SERPINI1,ETFDH,CASK
GO:0009889	Regulation ofBiosynthetic Process	PLK1,HHEX,MSX1,TCF21,HAT1,ANP32A,HIVEP2,OLIG1,PRKAA2,NFE2L2,SMARCD2,PNRC1,ING3,PSIP1,IKBKAP,MED4,ADRB3,SNCA,TF,NEUROD1,DDX5,PLA2G4A,TLR2,IGF1R,CASK,PLSCR1
GO:0009069	Serine Family AminoAcid Metabolic Process	SRR,PSPH,TXNRD1
GO:0031328	Positive Regulation ofCellular BiosyntheticProcess	ADRB3,TF,NEUROD1,NFE2L2,DDX5,MED4,PLA2G4A,TLR2,IGF1R,CASK,HHEX,MSX1,PLSCR1,TCF21,PSIP1,SNCA

Note: GO.ID stands for the ID of gene ontology term. Term is the name of gene ontology term. Genes stand for the target genes associated with the GO.ID.

**Table 3:** Target genes and enriched GO terms in the molecular function ontology.

GO.ID	Term	Genes
GO:0005488	Binding	ALDH6A1,PLK1,IGF1R,ACTC1,CASK,UBE2G1,PRKAA2,SSB,CLCN3,DDX5,EIF4B,IRGM,SRR,LIG3,DRG1,PTBP2,MCCC2,SNCA,SCFD1,STX12,SLC6A1,SH3KBP1,APBB3,TLR2,MAP2K1IP1,RNASEH1,PSPH,NEUROD1,PLA2G4A,ANXA4,PLSCR1,GALNT3,MSX1,HIVEP2,SYCP1,H2AFZ,OLIG1,NFE2L2,IKBKAP,XRCC4,PSIP1,HHEX,KLHDC3,HDLBP,DUSP11,ANP32A,NLGN1,GDF10,TF,CYP2E1,PGCP,TDO2,ASPA,NLN,ETFDH,FBXO30,ING3,RIOK2,MGC108823,PLEKHA3,AMBN,EPB4.1L5,MED4,FCGR2B,ADRB3,CLTA,TXNRD1,TCF21,SEC61A1,CTBS,TTC12
GO:0042043	Neurexin FamilyProtein Binding	CASK,NLGN1
GO:0015179	L-amino AcidTransmembraneTransporter Activity	SLC6A1,SERINC1,SERINC3
GO:0046983	Protein DimerizationActivity	ADRB3,TXNRD1,HHEX,RNASEH1,SRR,PSPH,NEUROD1,CLCN3,TLR2,NFE2L2,NLGN1,TCF21
GO:0001105	RNA Polymerase IITranscription CoactivatorActivity	NEUROD1,PSIP1
GO:0001104	RNA Polymerase IITranscription CofactorActivity	NEUROD1,PSIP1,MED4
GO:0005515	Protein Binding	NEUROD1,APBB3,IGF1R,MSX1,SNCA,PRKAA2,ANP32A,NLGN1,GDF10,PLSCR1,CASK,DDX5,PLK1,XRCC4,AMBN,EPB4.1L5,HHEX,MED4,ACTC1,SH3KBP1,CYP2E1,FCGR2B,NFE2L2,SCFD1,CLCN3,SRR,UBE2G1,ADRB3,PSIP1,ING3,TXNRD1,RNASEH1,PSPH,TCF21,TLR2,STX12,SLC6A1,MAP2K1IP1
GO:0001190	RNA Polymerase IITranscription FactorBinding TranscriptionFactor Activity Involvedin Positive Regulation ofTranscription	NEUROD1,PSIP1
GO:0000166	Nucleotide Binding	PLK1,IGF1R,ACTC1,CASK,UBE2G1,PRKAA2,CLCN3,IKBKAP,DDX5,SRR,LIG3,RIOK2,MCCC2,IRGM,DRG1,MGC108823,CYP2E1,TXNRD1,SSB,EIF4B,PTBP2
GO:0015171	Amino Acid TransmembraneTransporter Activity	SLC6A1,SERINC1,SERINC3

**Table 4:** Target genes and enriched GO terms in the cellular component ontology.

GO.ID	Term	Genes
GO:0043231	intracellularmembrane-boundedorganelle	CYP2E1,TMCO1,IRGM,KLHDC3,SYCP1,PLK1,CTBS,TF,SNCA,TXNRD1,ALDH6A1,CROT,NLN,ETFDH,RNASEH1,MCCC2,PLA2G4A,ANP32A,SCFD1,SEC61A1,SERINC1,ADRB3,NEUROD1,CASK,HIVEP2,H2AFZ,OLIG1,HDLBP,PRKAA2,HHEX,ASPA,MSX1,SSB,NFE2L2,SMARCD2,SH3KBP1,PLSCR1,IKBKAP,TCF21,PNRC1,DDX5,HAT1,DUSP11,LIG3,DRG1,MED4,XRCC4,PTBP2,ING3,PSIP1,GALNT3,MAP2K1IP1,CLCN3,HERPUD1,KCNS3,SERINC3,YIPF2,CLTA,STX12
GO:0043227	membrane-boundedorganelle	CYP2E1,TMCO1,IRGM,KLHDC3,SYCP1,PLK1,CTBS,TF,SNCA,TXNRD1,ALDH6A1,CROT,NLN,ETFDH,RNASEH1,MCCC2,PLA2G4A,ANP32A,SCFD1,SEC61A1,SERINC1,ADRB3,NEUROD1,CASK,HIVEP2,H2AFZ,OLIG1,HDLBP,PRKAA2,HHEX,ASPA,MSX1,SSB,NFE2L2,SMARCD2,SH3KBP1,PLSCR1,IKBKAP,TCF21,PNRC1,DDX5,HAT1,DUSP11,LIG3,DRG1,MED4,XRCC4,PTBP2,ING3,PSIP1,GALNT3,MAP2K1IP1,CLCN3,HERPUD1,KCNS3,SERINC3,YIPF2,CLTA,STX12
GO:0044424	intracellular part	CYP2E1,TMCO1,IRGM,GNG10,PLK1,NFE2L2,H2AFZ,KLHDC3,SYCP1,SSB,DDX5,CTBS,TF,SNCA,TXNRD1,ALDH6A1,CROT,NLN,ETFDH,RNASEH1,MCCC2,PLA2G4A,CASK,PLSCR1,SPHK2,XRCC4,ANP32A,SCFD1,SEC61A1,SERINC1,ADRB3,NEUROD1,HIVEP2,OLIG1,HDLBP,PRKAA2,HHEX,ASPA,MSX1,SMARCD2,SH3KBP1,IKBKAP,TCF21,PNRC1,HAT1,DUSP11,LIG3,DRG1,MED4,PTBP2,ING3,PSIP1,GALNT3,ACTC1,ANXA4,KCNS3,APBB3,SRR,PSPH,EPB4.1L5,TLR2,MAP2K1IP1,CLCN3,HERPUD1,SERINC3,YIPF2,CCT6A,NLGN1,CLTA,STX12
GO:0000267	cell fraction	PLA2G4A,ADRB3,SNCA,CASK,HRH4,SPHK2,ACTC1,TDO2,CROT,DDX5,SRR,CYP2E1,IGF1R,SLC6A1,SH3KBP1,PSPH,SEC61A1
GO:0043229	intracellular organelle	CYP2E1,TMCO1,IRGM,PLK1,NFE2L2,H2AFZ,KLHDC3,SYCP1,CTBS,TF,SNCA,TXNRD1,ALDH6A1,CROT,NLN,ETFDH,RNASEH1,MCCC2,PLA2G4A,ANP32A,SCFD1,SEC61A1,SERINC1,ADRB3,NEUROD1,CASK,HIVEP2,OLIG1,HDLBP,PRKAA2,HHEX,ASPA,MSX1,SSB,SMARCD2,SH3KBP1,PLSCR1,IKBKAP,TCF21,PNRC1,DDX5,HAT1,DUSP11,LIG3,DRG1,MED4,XRCC4,PTBP2,ING3,PSIP1,GALNT3,MAP2K1IP1,CLCN3,HERPUD1,KCNS3,SERINC3,YIPF2,ACTC1,EPB4.1L5,NLGN1,APBB3,CLTA,STX12
GO:0043226	organelle	CYP2E1,TMCO1,IRGM,PLK1,NFE2L2,H2AFZ,KLHDC3,SYCP1,CTBS,TF,SNCA,TXNRD1,ALDH6A1,CROT,NLN,ETFDH,RNASEH1,MCCC2,PLA2G4A,ANP32A,SCFD1,SEC61A1,SERINC1,ADRB3,NEUROD1,CASK,HIVEP2,OLIG1,HDLBP,PRKAA2,HHEX,ASPA,MSX1,SSB,SMARCD2,SH3KBP1,PLSCR1,IKBKAP,TCF21,PNRC1,DDX5,HAT1,DUSP11,LIG3,DRG1,MED4,XRCC4,PTBP2,ING3,PSIP1,GALNT3,MAP2K1IP1,CLCN3,HERPUD1,KCNS3,SERINC3,YIPF2,ACTC1,EPB4.1L5,NLGN1,APBB3,CLTA,STX12
GO:0030139	endocytic vesicle	CLTA,IRGM,STX12,TF
GO:0005634	nucleus	KLHDC3,SYCP1,PLK1,CASK,PRKAA2,PSIP1,MSX1,PTBP2,TXNRD1,PLSCR1,IKBKAP,XRCC4,ANP32A,HAT1,SMARCD2,MED4,ING3,DDX5,PLA2G4A,ADRB3,SNCA,NEUROD1,HIVEP2,H2AFZ,OLIG1,HDLBP,HHEX,ASPA,ALDH6A1,SSB,NFE2L2,SH3KBP1,TCF21,PNRC1,DUSP11,RNASEH1,LIG3,DRG1,GALNT3
GO:0034399	nuclear periphery	CASK,ANP32A,HAT1,PSIP1
GO:0019717	synaptosome	SNCA,CASK,SLC6A1,SH3KBP1,PSPH

**Table 5:** Number of target genes involved in the biological process (BP) ontology.

			Number of target genes regulated by each microRNA									
Term (GO.ID)	EnrichmentScore	Count	miR -144-3p	miR -340-5p	miR -186-5p	miR -153-3p	miR -132-3p	miR -674-5p	let-7b-5p	miR -190a-5p	miR -142-3p	miR -138-1-3p
nitrogen compoundmetabolic process(GO:0006807)	4.238180	42	10	10	5	5	5	1	2	2	1	1
response to organicsubstance (GO:0010033)	3.806089	26	8	4	3	4	3	2		1	1	
cellular nitrogen compoundmetabolic process(GO:0034641)	3.691296	40	10	10	5	4	5	1	2	1	1	1
positive regulationof cellular process(GO:0048522)	3.582448	32	7	8	4	4	3	2	1	2	1	
regulation of cellularbiosynthetic process(GO:0031326)	3.356312	28	8	7	2	3	2	1	2	1	1	1
response to stress(GO:0006950)	3.283229	27	8	6	4	2	2	3	1		1	
primary metabolicprocess (GO:0044238)	3.271385	59	18	13	7	6	6	2	2	2	2	1
regulation of biosyntheticprocess (GO:0009889)	3.245952	28	8	7	2	3	2	1	2	1	1	1
serine family amino acidmetabolic process(GO:0009069)	3.235984	3	1		1		1					
positive regulationof cellular biosyntheticprocess (GO:0031328)	3.157633	18	6	4	1	2	1	1	1	1	1	
		Total	84	69	34	33	30	14	13	11	10	5

**Table 6:** Number of target genes involved in the molecular function (MF) ontology.

			Number of target genes regulated by each microRNA involved in MF terms									
Term (GO.ID)	EnrichmentScore	Count	miR -144-3p	miR -340-5p	miR -186-5p	miR -132-3p	miR -190a-5p	miR -153-3p	miR -142-3p	miR -674-5p	let-7b-5p	miR -138-1-3p
binding (GO:0005488)	3.525446	76	18	17	12	9	5	5	2	4	3	1
neurexin family proteinbinding (GO:0042043)	2.812533	3	1				1		1			
L-amino acidtransmembrane transporter activity (GO:0015179)	2.792501	3			1	1			1			
protein dimerizationactivity (GO:0046983)	2.638246	13	4	1	3	1	2	1			1	
RNA polymerase IItranscription coactivatoractivity (GO:0001105)	2.344007	2		1			1					
RNA polymerase IItranscription cofactoractivity (GO:0001104)	2.342288	3	1	1			1					
protein binding(GO:0005515)	2.293113	41	10	9	5	4	4	3	2	2	2	
RNA polymerase IItranscription factor bindingtranscription factor activityinvolved in positiveregulation of transcription(GO:0001190)	2.162053	2		1			1					
nucleotide binding(GO:0000166)	2.150798	22	6	4	5	3		1	1	1	1	
amino acid transmembranetransporter activity(GO:0015171)	2.125126	3			1	1			1			
		Total	40	34	27	19	15	10	8	7	7	1

**Table 7:** Number of target genes involved in the cellular component (CC) ontology.

			Number of target genes regulated by each microRNA involved in CC terms									
Term (GO.ID)	Enrichment Score	Count	miR -340-5p	miR -144-3p	miR -186-5p	miR -132-3p	miR -153-3p	miR -674-5p	miR -142-3p	miR -190a-5p	let-7b-5p	miR -138-1-3p
intracellularmembrane-boundedorganelle(GO:0043231)	4.113860	64	15	14	10	7	6	4	3	2	2	1
membrane-boundedorganelle(GO:0043227)	4.077967	64	15	14	10	7	6	4	3	2	2	1
intracellular part(GO:0044424)	3.672762	75	16	18	11	8	7	4	3	4	3	1
cell fraction(GO:0000267)	3.437629	19	5	3	1	2	3	3	1		1	
intracellular organelle(GO:0043229)	3.398168	68	16	14	10	7	6	4	3	4	3	1
organelle(GO:0043226)	3.347499	68	16	14	10	7	6	4	3	4	3	1
endocytic vesicle(GO:0030139)	3.218764	4			1	1		1	1			
nucleus(GO:0005634)	3.173304	41	12	10	6	5	3		1	1	2	1
nuclear periphery(GO:0034399)	3.103823	5	1	2		1			1			
synaptosome(GO:0019717)	2.862995	6	1	1	1	1	1		1			
		Total	97	90	60	46	38	24	20	17	16	6

### Assessment of miRNA by qRT-PCR

**Figure 5:**
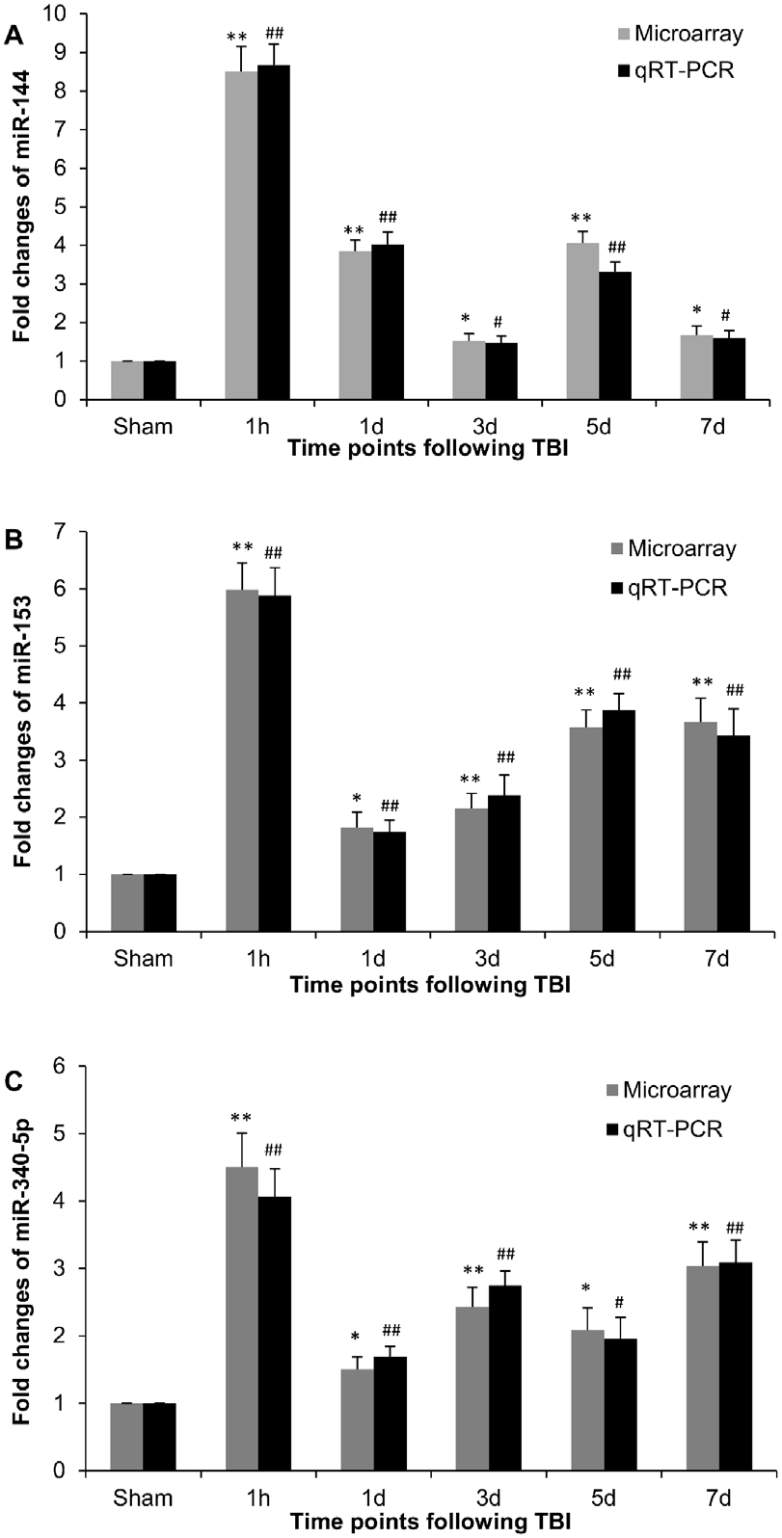
Expression levels of miR-144 (A), miR-153 (B) and miR-340-5p (C) assessed by miRNA array and miRNA-specific qRT-PCR. Ipsilateral hippocampi collected from sham and TBI rats were used to extract total RNA for both assays. Data are means ± SEM. N = 5. *P<0.05 and **P<0.01 *vs.* sham group for miRNA array data. #*P*<0.05 and ##*P*<0.01 *vs.* sham group for qRT-PCR results.

Three selected miRNAs (miR-144, -153 and -340-5p) were detected by qRT-PCR using TaqMan MicroRNA Assays to validate the microarray results. The primers and probes specific to miR-144, -153 and -340-5p (Invitrogen, Life Technologies) are shown in table 8. U6 served as internal control. qRT-PCR results showed that expression levels of miR-144, -153 and -340-5p in ipsilateral hippocampus were significantly elevated at all five time points after TBI, compared with that of sham controls (Figure 5). The expression patterns were in complete agreement with the miRNA array data. There is a very strong correlation between miRNA array and qRT-PCR results for miR-144 (*R* ^2^ = 0.984, *P*<0.001), miR-153 (*R* ^2^ = 0.982, *P*<0.001) and miR-340-5p (*R* ^2^ = 0.958, *P*<0.001).

**Table 8:** Primers for miRNA-specific qRT-PCR.

Purpose	miRNA	Primer Sequence
ReverseTranscriptaseReaction		
	U6	5′-CGCTTCACGAATTTGCGTGTCAT-3′
	rno-miR-144	5′-GTCGTATCCAGTGCGTGTCGTGGAGTCGGCAATTGCACTGGATACGACAGTACA-3′
	rno-miR-340-5p	5′-GTCGTATCCAGTGCGTGTCGTGGAGTCGGCAATTGCACTGGATACGACAATCAG-3′
	rno-miR-153	5′-GTCGTATCCAGTGCGTGTCGTGGAGTCGGCAATTGCACTGGATACGACGATCAC-3′
Quantitativereal-time PCR		
	U6	F: 5′-GCTTCGGCAGCACATATACTAAAAT-3′ R: 5′-CGCTTCACGAATTTGCGTGTCAT-3′
	rno-miR-144	GSP: 5′-GGGGGTACAGTATAGATGAT-3′ R: 5′-TGCGTGTCGTGGAGTC-3′
	rno-miR-340-5p	GSP: 5′-GCGGTTATAAAGCAATGAGA-3′ R: 5′-GTGCGTGTCGTGGAGTCG-3′
	rno-miR-153	GSP: 5′-GGGGGTTGCATAGTCACAAAA-3′ R: 5′-GTGCGTGTCGTGGAGTCG-3′

Note: GSP, gene specific primer; F, forward primer; R, reverse primer.

### Correlation between miR-144, miR-153 and miR-340-5p

**Figure 6:**
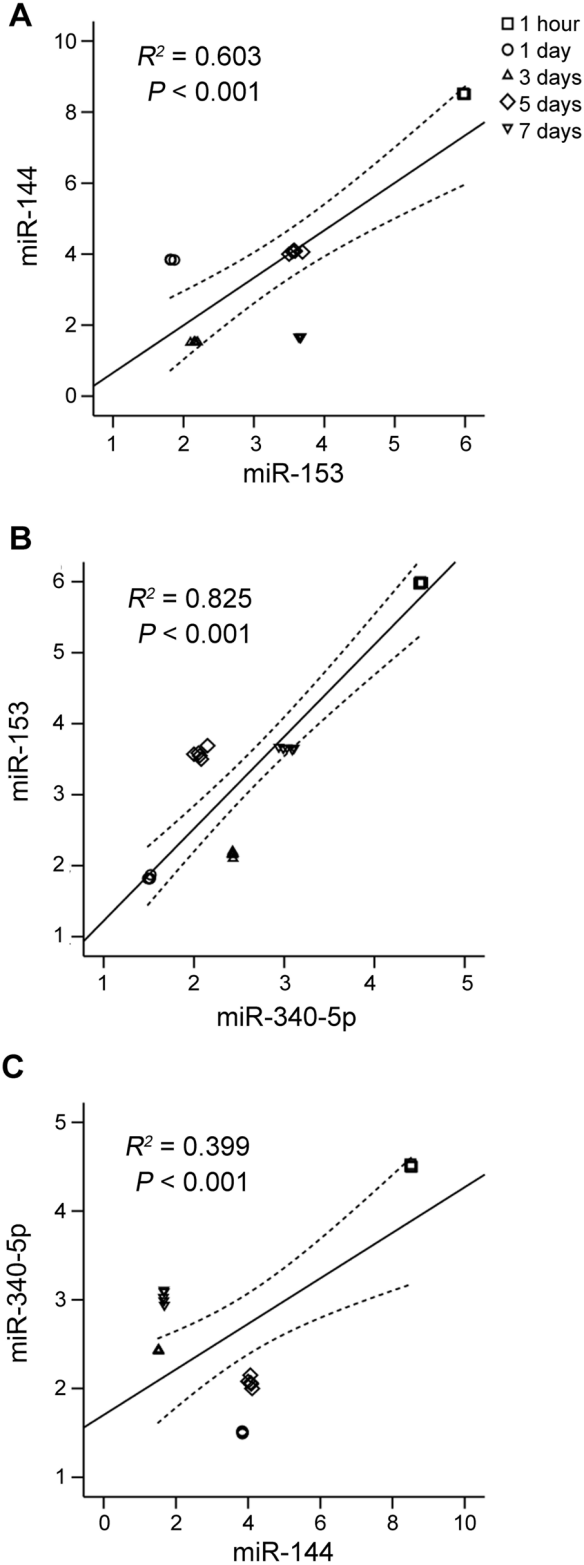
Correlation analyses between miR-144 and miR-153 (A), miR-153 and miR-340-5p (B) or miR-144 and miR-340-5p (C). Hippocampal miRNA levels from sham and all TBI rats were used for correlation analysis. The *R^2^* represents correlation coefficient. The correlation is significant at the 0.01 level (2-tailed).

Since their expression patterns after TBI were similar (Figure 5), correlation analyses were carried out for miR-144, -153 and -340-5p. The correlation analyses showed that miR-144, miR-153 and miR-340-5p have positive interrelated relationships between each other (Figure 6), among which the highest correlation coefficient was obtained between miR-153 and miR-340-5p (*R^2^* = 0.825, *P*<0.001) (Figure 6B).

### Protein Expression of Selected Target Genes in Ipsilateral Hippocampus

**Figure 7:**
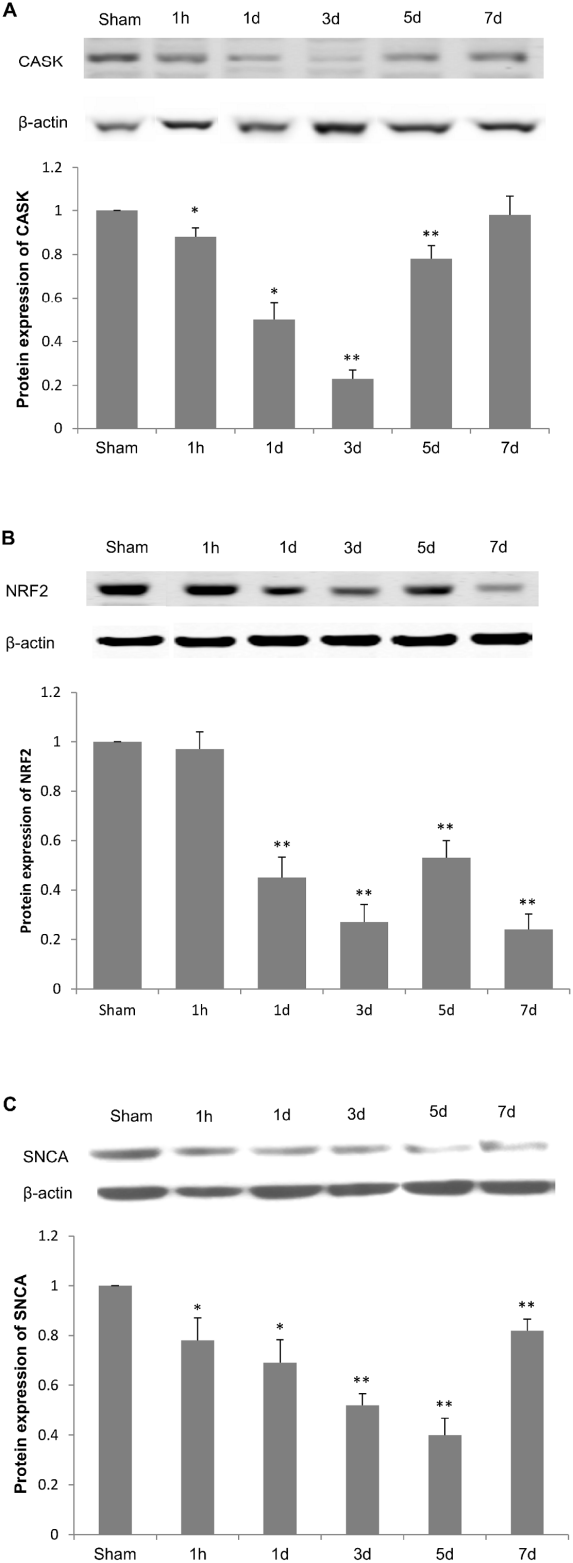
Protein expression of CASK, NRF2 and SNCA in rat hippocampus after TBI. Western blots were used to detect CASK (A), NRF2 (B) and SNCA (C) protein levels in whole cell lysates of hippocampus. Relative protein expression levels were determined by normalizing band density of target protein to that of β-Actin and comparing with sham group. Data are presented as mean ± SEM. N = 5. *P<0.05 and **P<0.01 *vs.* sham group.

Among the predicted target genes, *Cask*, *Nfe212* and *Snca* were selected for further investigation because of their involvement in synaptic dysfunction and cognitive disorders [21]–[25]. We examined protein expression levels in whole tissue lysates of ipsilateral hippocampi from all 6 groups of rats. Western blot results showed a significant decrease in the protein expression of CASK in hippocampus at 1 hour, 1 day and 3 days post TBI when compared to sham-operated group, and this decrease began to recover at 5 days and 7 days post TBI (Figure 7A).

We also detected the alteration in expression of NRF2, the protein of *Nfe212* gene which is a putative target of both miR-144 and miR-153, in hippocampus post TBI. We observed a consistent reduction of NRF2 from 1 day to 7 days post injury compared with that in sham group (Figure 7B). There was no significant reduction of NRF2 at 1 hour post injury.

SNCA, one of the target proteins of miR-153, was also significantly suppressed in hippocampus after TBI (Figure 7C). The SNCA protein level began to decrease at 1 hour post TBI, and reached the lowest level at 5 days post TBI. On day 7 after injury, the expression of SNCA began to recover but it was still less than the normal level.

### Histological Changes of Hippocampus after TBI

**Figure 8:**
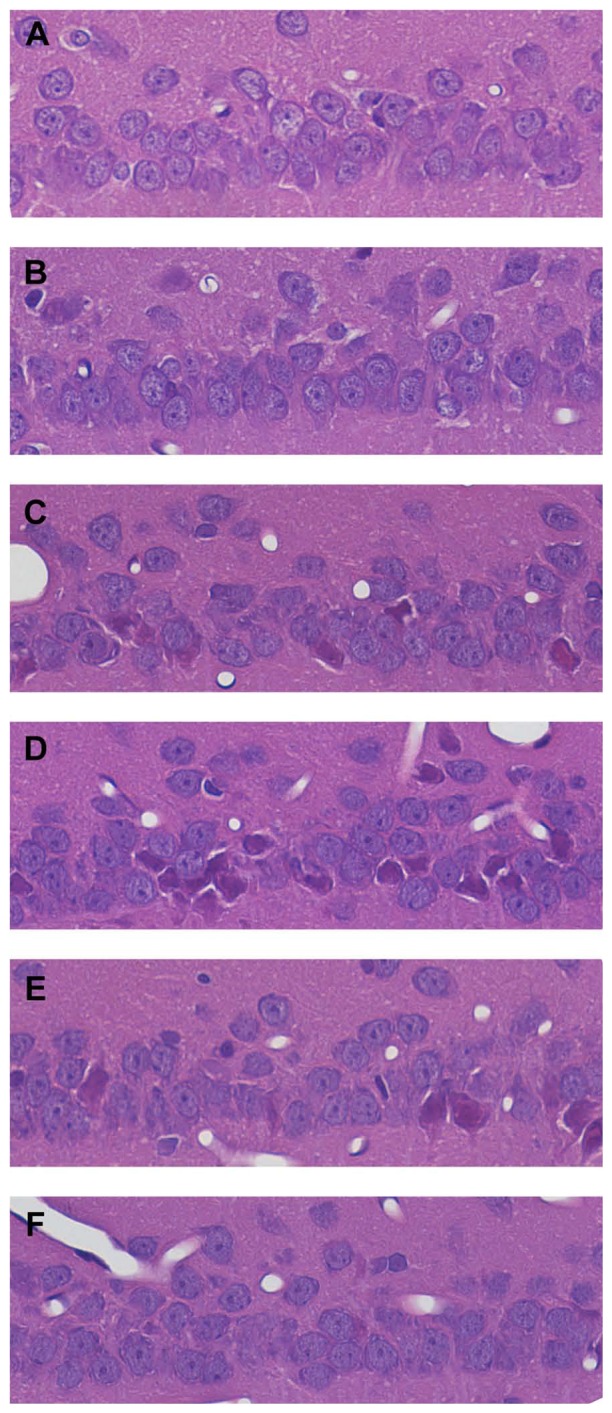
Representatives of hippocampal cross sections stained with hematoxylin and eosin (400X). CA1(A) Sham-operated. (B) One-hour post TBI. (C) One-day after TBI. (D) Three-day post TBI. (E) Five-day after TBI. (F) Seven-day post TBI.

Histological changes at different time points post TBI were assessed by H&E staining. There was no significant lesion or cell death in the hippocampi of sham group (Figure 8A). Neurons had round and pale-staining nuclei with prominent nucleoli. In contrast, neuronal cell death was observed at 1 hour after TBI and it increased time-dependently from 1 hour to 3 days post TBI (Figure 8B to 8D). Neurons were arranged loosely and confusedly with shrinkage and irregular cell shape (Figure 8C, Figure 8D). The boundaries between karyotheca and cytoplasm were blurred and, eventually, dissolved. Characteristic morphological changes of apoptosis were observed in some hippocampal neurons, demonstrated as the nucleolus disappearance. On day 5 post injury, nuclear membrane became gradually clear and complete, and basic nuclear chromatin showed a normal distribution with sporadic dying neurons (Figure 8E). Seven days post injury, the hippocampus structures were almost complete and neurons returned to normal (Figure 8F).

### TBI-induced Neuronal Apoptosis in Hippocampus

**Figure 9:**
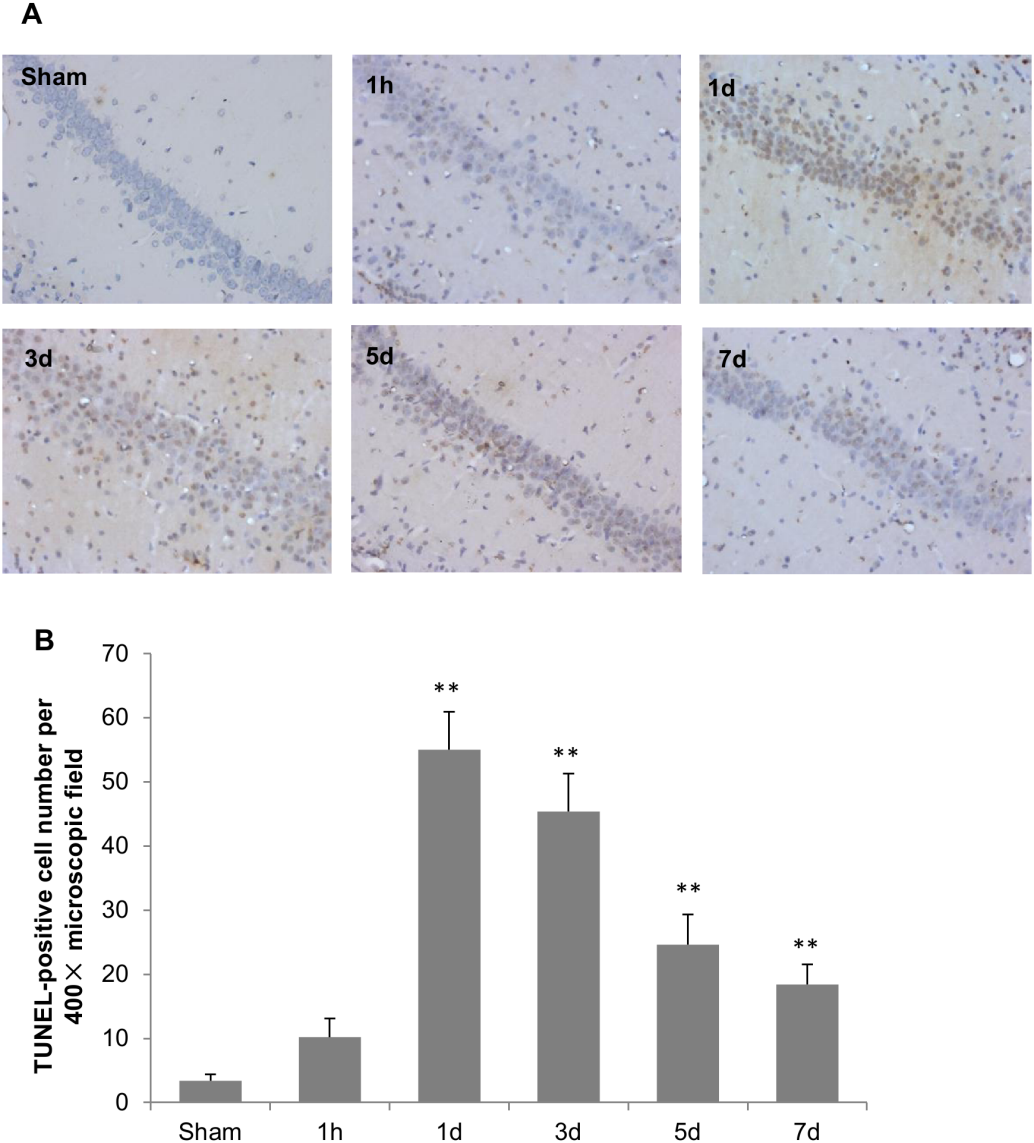
Histopathological analysis of TBI-induced apoptosis. (A) TUNEL staining of the hippocampal CA1 section of sham and TBI rats (200X). (B) Average numbers of TUNEL-positive cells in hippocampal CA1 section. The stained cells were counted in four microscopic fields of each slide at a 400X magnification. Data represent means ± SEM. N = 5. *P<0.05 and **P<0.01 *vs.* sham group.

Very few TUNEL-positive cells were detected in the hippocampus CA1 subfield in the sham group (Figure 9A, upper left panel). Increased apoptotic cells were observed one hour after TBI. The number of TUNEL-positive cells reached the peak 1 day after TBI and gradually declined thereafter. On day 3, the percentage of apoptotic neurons remained high and the number of apoptotic cells was still significantly greater at 7 days post TBI, compared with that in sham controls (Figure 9).

**Figure 10:**
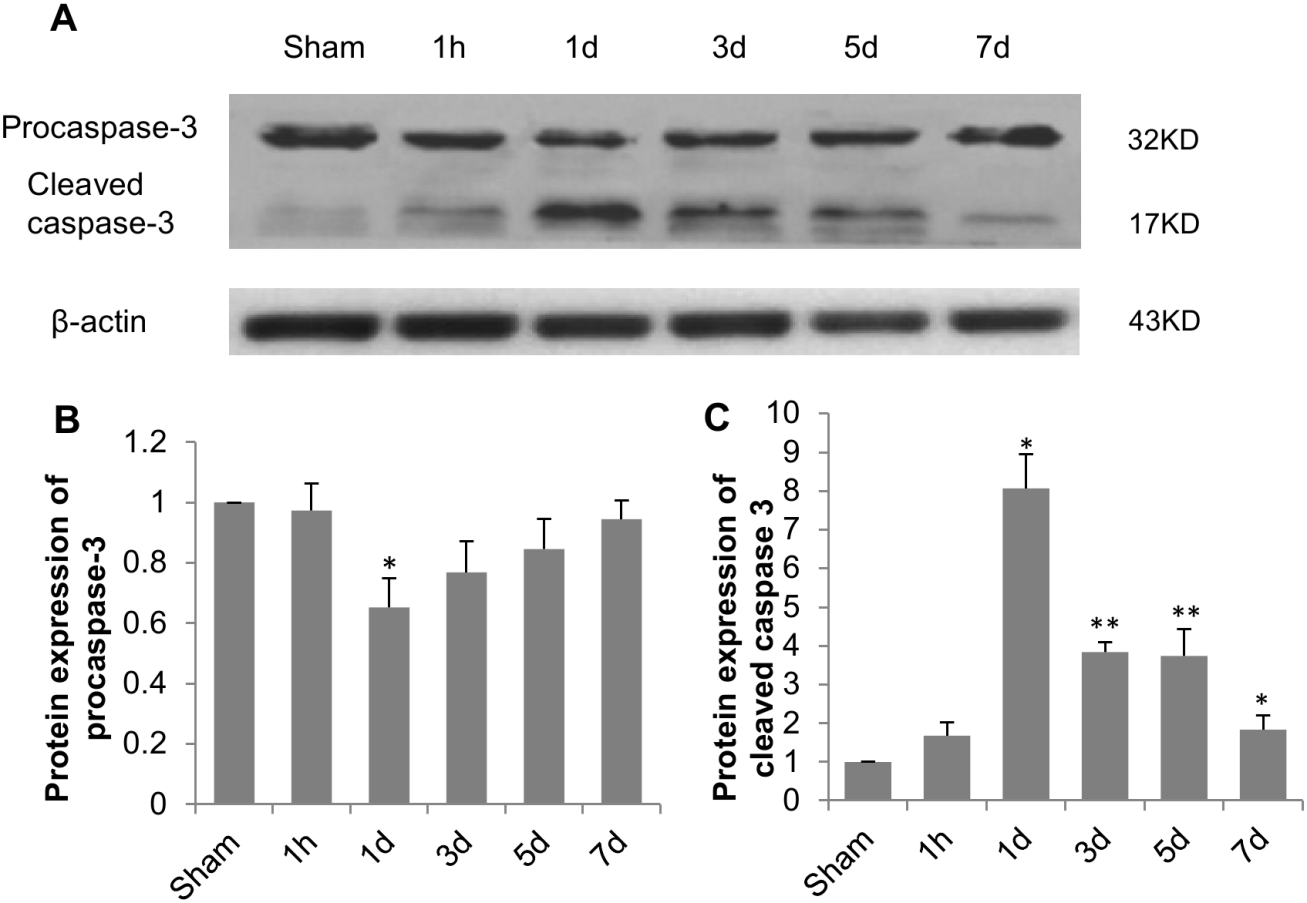
Caspase 3 activation in rat hippocampus after TBI. (A) Western blot analysis of procaspase 3 and cleaved caspase 3 in hippocampus. β-actin served as loading control. Relative protein expression levels were determined by normalizing band density of procaspase 3 (B) or cleaved caspase 3 (C) to that of β-Actin and comparing with sham group. Data are presented as mean ± SEM. N = 5. *P<0.05 and **P<0.01 *vs.* sham group.

To quantitatively examine the kinetics of apoptosis induced by TBI, caspase 3 activation was assessed by western blotting. Our results showed that TBI markedly activated procaspase 3 in hippocampus one day after the injury (Figure 10), evidenced by suppression of procaspase 3 and significant induction of cleaved caspase 3. After day 3, the protein level of active caspase 3 dropped with time, but remained higher than sham group 7 days post TBI.

### Synapse Ultrastructure after TBI

**Figure 11:**
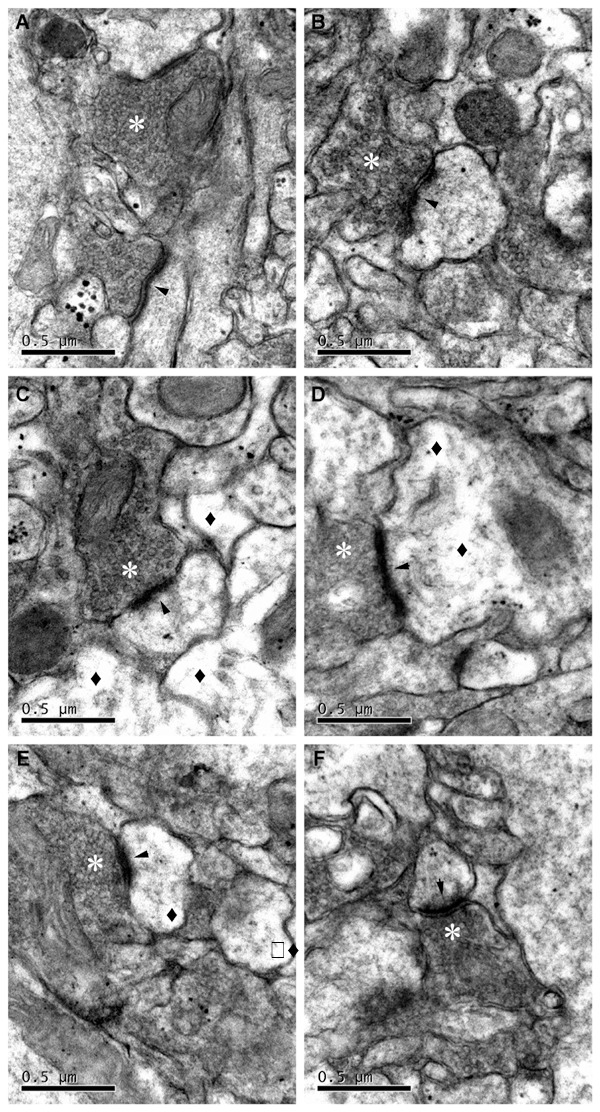
Synapse ultrastructure in hippocampi of sham and TBI rats. The synapse ultrastructure in CA1(A) Sham-operated. (B) One-hour post TBI. (C) One-day after TBI. (D) Three-day post TBI. (E) Five-day after TBI. (F) Seven-day post TBI. Scale bars = 0.5 µm. * synaptic vesicles density, ▴synaptic cleft, ♦vacuolation.

Electron microscopic examination at 37,000x magnifications revealed unambiguous synaptic structures (Figure 11). In the sham operation group (Figure 11A), the synaptic structure in CA1 section of the hippocampus was normal. The mitochondria envelope and cristae appeared intact. Synaptic vesicles in the presynaptic bouton were clearly visible. The front and back membranes of the synapses were clear. By contrast, the membranes of the synapses became illegible in TBI groups. At the early stage of TBI from 1 hour to 3 days post injury, hazy vesicles, swelling of neurons, dissolution of microtubules and filaments, blurred synaptic cleft, thinner postsynaptic density (PSD) and shorter length of synaptic active zone (SAZ) were observed (Figure 11B–D). On the 5^th^ and 7^th^ day post-injury (Figure 11E, F), the swelling of nerve fibers was reduced and the cell morphology was basically restored, and all the synaptic structural parameters were gradually normalized.

## Discussion

TBI remains a major public health problem, and little can be done to reverse the initial brain damage caused by trauma. To develop intervention strategies to limit the primary and secondary brain damage that occurs within days of a head trauma, more studies need to be done to decipher the biological mechanisms underlying damage to the brain, such as changes in miRNAs that are enriched in the brain and might mediate secondary brain damage. Previous studies have demonstrated that TBI induces extensive temporal changes in the cerebral mRNA, protein and miRNA expression [15], [16], [19]. In the present study, we examined the dynamic changes of miRNA transcriptome, histological changes, apoptotic alterations, synapse ultrastructure and protein expression in rat hippocampus at five time points (1-hr, 1-day, 3-day, 5-day and 7-day) after injury. The results showed that a large set of miRNAs in the ipsilateral hippocampus were significantly dysregulated after TBI, many of which were not identified in previous reports [15], [16].

We found that miRNAs respond rapidly to TBI. In fact, vast majority of the miRNAs gained the most fold changes at one hour post TBI (table 1). While the expression levels of some miRNAs sustained until day 7 post TBI, some miRNAs gradually declined. It is noteworthy that each time point post TBI featured a completely diverse and unique miRNA expression profile. On the other hand, both up- and down-regulation of some miRNAs are detected at different time points in the injured hippocampus following TBI. The number and expression levels of altered miRNAs at different time points varied greatly, probably due to different regulatory mechanisms and stabilities, and the unique miRNA expression profile might be potentially used as molecular markers in injured hippocampus for evaluation of TBI progress.

GO analysis of selected miRNAs (miR-142-3p, -144, -340-5p, -674-5p, -153, -186, -190, -132*, -138-1* and let-7b) revealed that many of their target genes are involved in biological processes and cellular functions that might be related to TBI pathophysiology. Further analysis showed that miR-144 and miR-340-5p share a common target gene *Etfdh*. While *Nfe212* and *Gdf10* are putative targets of miR-144 and miR-153, *Crot* and *Plekha3* are potentially regulated by miR-153 and miR-340-5p, which compelled us to validate the expression of these three miRNAs by miRNA-specific qRT-PCR. Interestingly, we found strong positive correlations among the expression patterns of miR-144, miR-153 and miR-340-5p in hippocampus after TBI. miR-144 and miR-340-5p are located in chromosome 10, whereas miR-153 is found in chromosome 6. There is no significant similarity found among these 3 miRNA sequences. In consideration of their correlations with craniocerebral diseases in literature, as well as the involvement of their target genes in cellular component of synaptosome, we would hypothesize that miRNA-miRNA interregulation/interaction among miR-144, miR-153 and miR-340-5p play important roles in the pathophysiological progress of brain injury, especially the development of cognitive dysfunction.

miR-144 is well known for its consistent increase in the aging brain, which is usually accompanied by a continuous cognitive decline, and miR-144 deregulation is considered a risk factor for disease development of Alzheimer's disease (AD)[26]. It plays a central coordinating role in the posttranscriptional regulation of genes such as Ataxin 1 (ATXN1) in the aging brain. miR-144 is overexpressed in AD patients and it may play a mechanistic role in AD pathogenesis via inhibition of ADAM10 protein[26], [27]. However, whether miR-144 is involved in TBI-induced cognitive disorder remains unknown. The present study, for the first time, demonstrated consistent up-regulation of miR-144 in the hippocampus at several time points up to 7 days post TBI. Bioinformatics analysis showed 25 potential target genes of miR-144, among which *Cask*, *Etfdh*, *Nfe212* and *Gdf10* garnered our attention because these genes are predicated targets of other altered miRNAs as well.

CASK, known as *Lin-2* in *C. elegans*, is a member of membrane-associated guanylate kinase (MAGUK) protein family. CASK is abundant in the nervous system and is a scaffolding protein of the active zone (AZ)[28], [29]. Its binding to partner proteins in different regions of the brain is involved in specific functions[28]–[30], including synapse formation and plasticity, regulation of ion channels at both presynaptic and postsynaptic junctions, gene expression and neural development[22], [31]–[34]. GO analysis in our study also indicated that CASK is involved in the cellular structure of synaptosome. In recent years, synaptic transmission and plasticity have been documented to correlate with memory and learning, and synaptic loss is pathologically correlated with cognitive impairment [35]–[37]. We hypothesized that CASK’s involvement in the synapse might underlie TBI-induced cognitive dysfunction. As expected, a significant decrease in CASK protein expression was found in hippocampus after TBI. The reduction dipped to the lowest level at 3-day post injury, and began to recover on day 5 post injury.

*Nfe212*, another predicted target gene of miR-144, is known for its anti-oxidation trait. NRF2, the protein of the *Nfe212* gene, declines with age. The NRF2 antioxidant signaling pathways have been identified as a promising therapeutic target for cognitive deficits in aging and neurodegenerative diseases [21], [25], [38]. Therefore, we investigated the protein expression of NRF2 in the hippocampus after TBI and found a consistent decrease in NRF2 expression at all five time points post TBI, indicating that TBI-induced cognitive dysfunction might be mediated, at least partially, via miR-144/NRF2 pathway.

Another notable miRNA in this study is miR-153 which has been demonstrated to be widely involved in the pathological process of many diseases with cognitive function impairment, such as Parkinson disease (PD) [39]–[41], AD [42], and some types of tumors[43]–[45]. Our miRNA arrays and qRT-PCR results showed that miR-153 was consistently overexpressed in hippocampus following TBI. Some predicted target genes of miR-153 such as *Nfe2l2*, *Crot*, *Gdf10*, *Sphk2* and especially *Snca*, are primarily involved in the GO terms that are closely related to the regulation of cellular process activity, interacting selectively and non-covalently with neurexins, synaptic cell surface proteins.

The alpha-synuclein (SNCA) protein is encoded by the *Snca* gene, which is a predicted target gene of miR-153 and has been documented to be post-transcriptionally regulated by miR-153[39]. SNCA is abundant in presynaptic terminals [46]–[50] and overexpression of SNCA can induce synaptic dysfunctions such as inhibited neurotransmitter release and reduced synaptic vesicle density [24], [51]. SNCA plays critical role in the neurodegenerative processes of PD and some specific types of AD [39]–[41], [52], [53]. Most patients with SNCA multiplication develop cognitive dysfunction in the early stage of PD [23]. The concentration of alpha-synuclein in cerebrospinal fluid (CSF) was markedly increased in patients subjected to TBI, and the increase is likely due to the release of soluble alpha-synuclein from the injured neurons and glial cells, and result of synaptic dysfunction and altered transport mechanisms following TBI [54], [55]. In mice, alpha-synuclein has been shown to transiently elevate in the axonal swellings and bulb in the traumatized brain of aged but not young rats after experimental TBI [56]. Thus, we tested the hypothesis that SNCA is dysregulated in rat hippocampus after TBI. Our results demonstrated gradual reduction of hippocampal SNCA from one hour to five days after TBI, concurrent with the increase of miR-153. We also noticed that SNCA was partially restored one week after TBI. This might be explained by the younger age of the rats used in our study. Aforementioned study in mice showed no change of hippocampal SNCA in young mice one week after injury, but no earlier time points were examined [56].

In addition to miR-144 and miR-153, miR-340-5p was consistently up-regulated in hippocampus post TBI. GO analysis showed that SH3KBP1, a target of miR-340-5p, is involved in cellular pathway of synaptosome, suggesting that miR-340-5p might also play a role in the pathophysiological process of TBI and TBI-induced cognitive functions. miR-340-5p is a widespread miRNA and is expressed in various organs of the body [57]–[60]. It was found differentially expressed in cerebral cortex of rats subjected to ischemic pre-conditioning [58], and in L_4_-L_6_ spinal segments after denervation injury [61]. However, research into miR-340-5p is still in its infancy, further studies are warranted.

We used both male and female rats in the present study and found no significant differences between males and females in miRNA expression profile and behavior. Sex hormones may affect outcome after TBI, but gender has simply not been previously considered to be an important risk factor in TBI outcome. Neuroprotective effects of estrogens have been suggested by most of the available data related to ischemic brain injury [62], although Soustiel and colleagues also reported the potential neuroprotective value of estrogens in TBI [63]. In another experimental brain trauma study, however, the authors found a protective effect of estrogen only in male rats [64]. Interestingly, progesterone has also been shown to have a protective effect in reducing brain edema in TBI sequelae in female rats [65]. In a mice study, gender and estrogen manipulation were found to have no effect on TBI [66]. There is no simple explanation for the disparities among the effects of gender in these studies. Further studies are needed to assess the impact of estrogen on TBI.

Taken together, we characterized the expression profiles of hippocampal miRNAs at five time points post TBI. While different time points have diverse and unique miRNA expression profile, 10 out of 156 reliably detected miRNAs were consistently up-regulated or down-regulated at all five time points. The potential target genes of those 10 miRNAs in hippocampus might be involved in various TBI pathophysiological processes. qRT-PCR analysis further confirmed that elevated expression of miR-144, miR-153 and miR-340-5p was rapid and long-lasting. In addition, protein levels of CASK, NRF2 and SNCA, which are putative targets of miR-144, miR-153 and miR-340-5p, were found to be consistently suppressed in hippocampus after TBI. Our data suggest that miR-144, miR-153 and miR-340-5p could be potential targets for diagnostic and therapeutic purposes against TBI.
